# A retrospective study of differential prognostic factors in early-onset *versus* late-onset colorectal cancer: a comprehensive clinical and machine learning analysis

**DOI:** 10.7717/peerj.21484

**Published:** 2026-07-01

**Authors:** Lifang Huang, Runtao Zhong, Kangkang Li, Jinfeng Chen, Yunxian He, Yuanning Ye, Shuxian Chen, Qili Wei, Huizhen Mai, Yali Zhang, Zhiqing Wang

**Affiliations:** 1 Department of Gastroenterology, Nanfang Hospital, Southern Medical University, Guangzhou, Guangdong, China; 2 Department of Gastroenterology, Zengcheng Central Hospital, Nanfang Hospital, Southern Medical University, Guangzhou, Guangdong, China; 3 Department of Health Management, Zengcheng Central Hospital, Nanfang Hospital, Southern Medical University, Guangzhou, Guangdong, China; 4 Department of Gastroenterology, Zengcheng Branch of Nanfang Hospital, Southern Medical University, Guangzhou, Guangdong, China

**Keywords:** Early-onset colorectal cancer, Late-onset colorectal cancer, Machine learning, Prognostic factors, LASSO regression, Random survival forest, Overall survival

## Abstract

**Background:**

The incidence of early-onset colorectal cancer (EO-CRC; age <50 years) has been increasing worldwide. This single-center retrospective study aimed to compare the clinical characteristics of EO-CRC and late-onset CRC (LO-CRC; age ≥ 50 years) and to identify age-specific prognostic factors for overall survival (OS).

**Methods:**

A total of 1,148 CRC patients were retrospectively analyzed and categorized into EO-CRC (*n* = 247) and LO-CRC (*n* = 901) groups. Clinical characteristics were compared using the Mann–Whitney U test and Chi-square test. Prognostic factors associated with OS were identified using Least absolute shrinkage and selection operator (LASSO) Cox regression followed by multivariate Cox modeling. Model performance was evaluated using the C-index, calibration curves, and time-dependent Receiver Operating Characteristic (ROC) analysis. Variable importance was further validated using a random survival forest (RSF) model.

**Results:**

EO-CRC patients showed higher proportions of family history, concurrent polyps, and Programmed Cell Death Ligand 1 (PD-L1) expression >10%, whereas LO-CRC patients exhibited higher rates of hypertension, diabetes, and elevated carcinoembryonic antigen (CEA) levels. Although OS did not differ significantly between groups (*P* = 0.460), their prognostic determinants varied markedly. In EO-CRC, distant metastasis, family history, Tumor, Node, and Metastasis (TNM) stage, PMS1 homolog 2, mismatch repair system component (PMS2), MutS Homolog 6 (MSH6), tumor size, concurrent polyps, and Ki-67 were major predictors. In LO-CRC, age, BRAF gene V600E mutation (BRAF V600E) mutation, elevated Carbohydrate antigen 19-9 (CA19-9), Ki-67, low hemoglobin, vascular invasion, MutL Homolog 1 (MLH1), and pathological type were significant contributors. The C-index values for the EO-CRC and LO-CRC models were 0.829 (SE = 0.023) and 0.751 (SE = 0.018), respectively, and all time-dependent ROC curves demonstrated Area Under the Curve (AUCs) above 0.70, indicating good predictive performance. RSF analyses further confirmed that distant metastasis and family history as the strongest predictors, while age and BRAF V600E are the strongest predictors for LO-CRC.

**Conclusion:**

This study suggests that EO-CRC and LO-CRC have fundamentally different prognostic determinants: the former emphasizes genetic susceptibility and tumor invasiveness, indicating that this group of patients may benefit from early genetic counseling, MMR/MSI testing, and immune checkpoint inhibitor therapy. The latter highlights age, acquired molecular changes, and chronic systemic factors, supporting the inclusion of metabolic and geriatric assessments in routine tumor care.

## Introduction

Colorectal cancer (CRC) remains a leading cause of cancer-related morbidity and mortality worldwide (Han et al., 2024; Bray et al., 2024). While overall CRC incidence has stabilized or declined in older populations, the incidence of early-onset CRC (EO-CRC) has been increasing by approximately 2% annually (Lang & Ciombor, 2022; Du et al., 2025). In the United States, the incidence rate of EO-CRC has been on the rise since the 1980s and 1990s (Siegel et al., 2023). From 1988 to 2015, the incidence rate of EO-CRC increased from 7.9 to 12.9 cases per 100,000 individuals, representing a 63% growth (Sinicrope, 2022). In stark contrast, the incidence rate in individuals over 50 years of age is declining (Siegel et al., 2023; Kanth & Inadomi, 2021). A study published by the Cancer Center of China in Journal of the National Cancer Center (JNCC) in 2024 indicated that in 2022, there were 517,000 new cases of colorectal cancer in China, ranking second among malignant tumors, and 240,000 deaths, ranking fourth (Han et al., 2024). Given the substantial burden of CRC in China, understanding age-specific prognostic determinants in the Chinese population is of critical clinical importance. Most EO-CRC cases are detected due to symptoms rather than through screening. The decline in incidence and mortality rates among individuals over 50 years of age (late-onset colorectal cancer, LO-CRC) is primarily attributed to the widespread implementation of screening programs (Du et al., 2025). Compared to patients with LO-CRC, those with EO-CRC experience a significantly longer time from symptom onset to diagnosis (Zaborowski et al., 2021). EO-CRC is often diagnosed at advanced stages and exhibits aggressive pathological features, including poor differentiation (mucinous adenocarcinoma 42.6% *vs.* 6.8%, medullary carcinoma 11.8% *vs.* 1.7%, *p* < 0.001) and higher rates of lymphatic (42.6% *vs.* 23.7%, *p* = 0.001) and vascular invasion (29.4% *vs.* 18.6%, *p* = 0.046) (Li et al., 2025; Hashmi et al., 2023). Despite the growing recognition of EO-CRC as a distinct clinical entity, existing studies have reported inconsistent findings regarding its prognostic factors compared with LO-CRC, largely due to differences in study design, sample composition, and analytical approaches. However, current evidence lacks age-specific prognostic models that integrate traditional statistical methods with machine learning algorithms. Moreover, the core differences in prognostic determinants between EO-CRC and LO-CRC remain to be elucidated.

Therefore, this study aims to address these deficiency by comprehensively comparing the clinical and molecular features between EO-CRC and LO-CRC, as well as constructing and validating age-specific prognostic models using traditional statistical and machine learning approaches.

## Methods

### Study population and data collection

A total of 1,148 CRC patients between 2018–2022 were diagnosed (This study has been granted an informed consent waiver by the Medical Ethics committee of NanFang Hospital of Southern Medical University: No. NFEC-2025-225. Patients were stratified into EO-CRC (<50 years, *n* = 247) and LO-CRC (≥50 years, *n* = 901). Inclusion criteria: (1) Diagnosed between 2018 and 2022; (2) Histopathological diagnosis. Exclusion criteria: (1) Pathological diagnosis TNM staging and differentiation degree are unknown; (2) Not undergoing surgery or chemotherapy; (3) Patients with malignant tumors of adjacent organs invading the colon; (4) Patients with malignant tumors of distant organs metastasizing to CRC; (5) Patients with recurrent CRC.

### Observation indicators and judgment criteria

Clinicopathological indicators: collect the gender, age, history of hypertension, history of diabetes, history of hyperlipidemia, weight, height, family history of colorectal cancer, combined polyps, hemoglobin, fecal occult blood CEA, CA19-9,CA72-4, remote metastasis, lymph node metastasis, histological grading, pathological classification, TNM staging, Ki-67%, PSM2, MLH1, Human Epidermal Growth Factor Receptor 2 (Her-2), MutS Homolog 2 (MSH2), MSH6, BRAF V600E, PD-L1 expression and other clinical pathological data and immunohistochemical features, among which body mass index (BMI) (18.5≥BMI<24.0 kg/m2; lean: BMI<18.5 kg/m2; overweight: 24≤BMI<28 kg/m2; obese BMI≥28.0 kg/m2). Anemia (Hb<120 g/L in adult males; Adult female (non pregnant) Hb<110 g/L), histological grading and pathological classification refer to the Nagtegaal et al. (2020) classification of digestive system tumors, divided into high, medium, and low differentiation; TNM staging adopts the AJCC 8th edition criteria, based on tumor infiltration depth (T), lymph node metastasis (N), and distant metastasis (M). Prognostic indicators: record the time and survival status of patients from diagnosis to death or the last follow-up.

### Statistical analysis

Multiple imputation was performed using the mice package in R under the assumption of missing at random. Five imputed datasets were generated with 10 iterations. Different imputation methods were applied according to variable types, including predictive mean matching for continuous variables, logistic regression for binary variables, and polytomous regression for categorical variables. Convergence of the imputation process was assessed using trace plots, and the distributions of observed and imputed values were compared to ensure plausibility. For subsequent analyses, one completed dataset was used for model development.

The normality of continuous variables was checked by Kolmogorov–Smirnov test. Since no variables follow the normality assumption, all continuous variables were reported with median and IQR while categorical variables were presented using number (n) and percentage (%). The comparison of medians between two groups was using Mann–Whitney U test. Categorical variables were compared using Chi-square test or Fisher’s exact text (if expected value ≤ 5 was found). A Kaplan–Meier survival analysis and log-rank test were used to compared the overall survival results between two groups.

Least Absolute Shrinkage and Selection Operator (LASSO) Cox proportional hazards regression was used to identify predictors of overall survival. Continuous variables were analyzed as numeric values, and categorical variables were dummy-encoded using the model.matrix() function. A right-censored survival object (Surv) was constructed with survival time in months and death coded as the event. Standardization of the continuous variables was automatically performed using the glmnet package (standardize = TRUE).

The LASSO approach applies an L1-penalty to shrink coefficients and perform variable selection. A 10-fold cross-validation procedure was used to determine the regularization parameter, and the lambda.1se value was selected to obtain a more parsimonious and robust model. The coefficient path was plotted to visualize shrinkage across penalty values, and variables with non-zero coefficients at *λ*1se were retained as important predictors.

The variables that are selected by LASSO as being significantly associated with OS will subsequently be confirmed using a multivariate Cox proportional hazards regression model. Here, the forward method and Wald test are employed to select the optimal combination of variables for predicting patient’s OS, with those that remain significant (*P* < 0.05) being the predictors. The predictive power of this multivariate model will be confirmed using the C-index, calibration plot, and time-dependent Receiver Operating Characteristic/Area Under the Curve (ROC/AUC). A nomogram is also built for each multivariate model. A C-index of 0.70–0.80 is generally considered to indicate good discriminative performance, whereas values above 0.80 suggest excellent discrimination.

A Random Survival Forest (RSF) model was used to explore the prognostic importance of the predictors being selected in the multivariate Cox model. RSF is a non-parametric ensemble learning method that extends the random forest algorithm to right-censored survival data. It does not require proportional hazards assumptions and can accommodate complex non-linear relationships and high-order interactions among variables. The RSF model was built using the randomForestSRC package in R with 1,000 trees. The mtry parameter (number of variables randomly selected at each node) was set to the default value (approximately the square root of the number of variables). Variable importance (VIMP) was calculated based on the decrease in prediction accuracy after permuting each variable, with larger VIMP values indicating greater prognostic contribution. The variables were ranked by their VIMP values, and a variable importance plot was generated to visualize the relative importance of each predictor.

All analyses were done using statistical software R (version 4.5.0; R Foundation for Statistical Computing, Vienna, Austria). The statistical significance level for all the tests was set at a *P* < 0.05, two-tailed.

## Results

### Data imputation

In all 1,148 patients, some variables had missing values, but the missing rates were all below 20%, and in fact, most were below 10% (Table S1). Therefore, we adopted multiple imputation and chose the corresponding method based on the type of the variables (continuous or categorical) to complete the imputation for subsequent analysis. The outcome variable and grouping variable have no missing values in the original data.

### Patient’s clinical characteristics

A total of 1,148 colorectal patients were included in this study, including 684 males and 464 females, their median age was 60 years. Among them, there are 247 individuals under the age of 50 (EO-CRC group), while the remaining 901 are aged 50 and above (LO-CRC group). Table 1 shows all the clinical characteristics and results of the patients grouped according to age.

**Table 1:** Patient’s clinical characteristics and results between age groups.

Parameters	EO-CRC (*n* = 247)	LO-CRC (*n* = 901)	All (*n* = 1148)	*P*
Sex				0.180
Male	138 (55.87%)	546 (60.60%)	684 (59.58%)	
Female	109 (44.13%)	355 (39.40%)	464 (40.42%)	
Age, year	43 (36, 47)	64 (57, 71)	60 (51, 69)	<0.001
Hypertension	31 (12.55%)	254 (28.19%)	285 (24.83%)	<0.001
Diabetes	18 (7.29%)	132 (14.65%)	150 (13.07%)	0.002
Hyperlipidemia	11 (4.45%)	32 (3.55%)	43 (3.75%)	0.508
Family history of colorectal cancer	30 (12.15%)	41 (4.55%)	71 (6.18%)	<0.001
BMI, kg/m2	22.39 (20.31, 24.77)	22.68 (20.57, 24.85)	22.66 (20.55, 24.84)	0.512
BMI group				0.835
Normal	132 (53.44%)	502 (55.72%)	634 (55.23%)	
Underweight	26 (10.53%)	79 (8.77%)	105 (9.15%)	
Overweight	71 (28.74%)	254 (28.19%)	325 (28.31%)	
Obesity	18 (7.29%)	66 (7.33%)	84 (7.32%)	
Overweight	89 (36.03%)	320 (35.52%)	409 (35.63%)	0.881
HGB, g/L	123 (98, 138)	120 (101, 133.50)	121 (101, 134)	0.043
Low HGB	91 (36.84%)	371 (41.18%)	462 (40.24%)	0.218
CEA	2.60 (1.29, 7.94)	3.44 (1.84, 8.98)	3.22 (1.73, 8.51)	0.001
CEA (+1 & log10 transformed)	0.56 (0.36, 0.95)	0.65 (0.45, 1.00)	0.63 (0.44, 0.98)	0.001
Elevated CEA	88 (35.63%)	349 (38.73%)	437 (38.07%)	0.373
CA19-9	9.29 (3.90, 22.34)	8.78 (4.17, 21.58)	8.91 (4.11, 21.75)	0.938
CA19-9 (+1 & log10 transformed)	1.01 (0.69, 1.37)	0.99 (0.71, 1.35)	1.00 (0.71, 1.36)	0.938
Elevated CA19-9	41 (16.60%)	144 (15.98%)	185 (16.11%)	0.815
CA72-4	1.77 (1.02, 4.28)	1.96 (1.05, 4.45)	1.92 (1.04, 4.44)	0.689
CA72-4 (+1 & log10 transformed)	0.44 (0.31, 0.72)	0.47 (0.31, 0.74)	0.47 (0.31, 0.74)	0.689
Elevated CA72-4	44 (17.81%)	134 (14.87%)	178 (15.51%)	0.258
Fecal occult blood	191 (77.33%)	754 (83.68%)	945 (82.32%)	0.020
Combined polyp	127 (51.42%)	395 (43.84%)	522 (45.47%)	0.034
Lesion				0.184
Rectum	19 (7.69%)	106 (11.76%)	125 (10.89%)	
Left half	159 (64.37%)	547 (60.71%)	706 (61.50%)	
Right half	69 (27.94%)	248 (27.52%)	317 (27.61%)	
Pathological type				<0.001
Ulcerative	78 (31.58%)	363 (40.29%)	441 (38.41%)	
Polypoid	133 (53.85%)	498 (55.27%)	631 (54.97%)	
Infiltrative	36 (14.57%)	40 (4.44%)	76 (6.62%)	
Maximum diameter of cancer				0.101
<5 cm	164 (66.40%)	547 (60.71%)	711 (61.93%)	
≥5 cm	83 (33.60%)	354 (39.29%)	437 (38.07%)	
Adenocarcinoma/high-grade epithelial neoplasia				0.918
Yes	212 (85.83%)	771 (85.57%)	983 (85.63%)	
No	35 (14.17%)	130 (14.43%)	165 (14.37%)	
Differentiation				<0.001
High	11 (4.45%)	31 (3.44%)	42 (3.66%)	
Medium	176 (71.26%)	742 (82.35%)	918 (79.97%)	
Low	60 (24.29%)	128 (14.21%)	188 (16.38%)	
Distant metastasis	42 (17.00%)	152 (16.87%)	194 (16.90%)	0.960
Nodal metastasis	97 (39.27%)	317 (35.18%)	414 (36.06%)	0.236
TNM stage				0.166
0–I–II	134 (54.25%)	444 (49.28%)	578 (50.35%)	
III–IV	113 (45.75%)	457 (50.72%)	570 (49.65%)	
Blood vessel				0.641
Negative	198 (80.16%)	710 (78.80%)	908 (79.09%)	
Positive	49 (19.84%)	191 (21.20%)	240 (20.91%)	
Lymphatic vessel				0.316
Negative	198 (80.16%)	747 (82.91%)	945 (82.32%)	
Positive	49 (19.84%)	154 (17.09%)	203 (17.68%)	
Nervous system				0.020
Negative	170 (68.83%)	547 (60.71%)	717 (62.46%)	
Positive	77 (31.17%)	354 (39.29%)	431 (37.54%)	
Her-2	144 (58.30%)	548 (60.82%)	692 (60.28%)	0.473
MLH1	219 (88.66%)	859 (95.34%)	1078 (93.90%)	<0.001
PMS2	207 (83.81%)	858 (95.23%)	1065 (92.77%)	<0.001
MSH2	228 (92.31%)	876 (97.23%)	1104 (96.17%)	<0.001
MSH6	216 (87.45%)	880 (97.67%)	1096 (95.47%)	<0.001
BRAF V600E	26 (10.53%)	118 (13.10%)	144 (12.54%)	0.280
PD-L1				0.015
≤10	159 (64.37%)	652 (72.36%)	811 (70.64%)	
>10	88 (35.63%)	249 (27.64%)	337 (29.36%)	
Ki-67				0.694
<80%	127 (51.42%)	476 (52.83%)	603 (52.53%)	
≥80%	120 (48.58%)	425 (47.17%)	545 (47.47%)	
Event				0.073
Survival	169 (68.42%)	668 (74.14%)	837 (72.91%)	
Dead	78 (31.58%)	233 (25.86%)	311 (27.09%)	

Based on the comparison results, LO-CRC have a higher proportion of hypertension, diabetes, fecal occult blood, ulcerative type, medium differentiation, nervous system positive, MLH1, PMS2, MSH2, MSH6, and higher levels of CEA; whereas EO-CRC has significantly higher rates of family history, HGB level, combined polyp, and PD-L1 >10.

**Figure 1:**
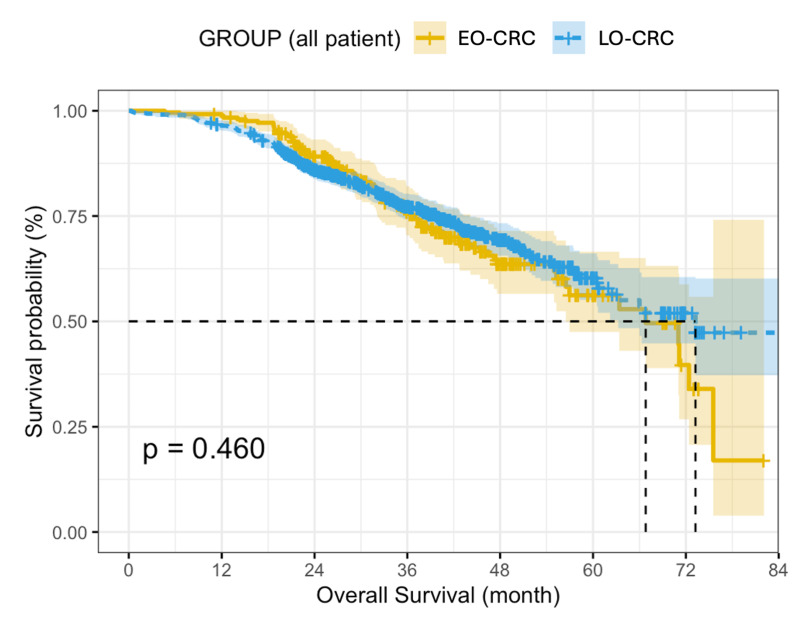
Kaplan–Meier overall survival (OS) curves comparing patients aged <50 years and ≥ 50 years.

However, there were no significant differences between the two groups in terms of the observed mortality rate during follow-up (31.58% *vs.* 25.86%, *P* = 0.073) or the overall survival (OS) outcomes predicted by Kaplan–Meier survival analysis (Fig. 1, *P* = 0.460). The following analysis will separate individuals EO-CRC and LO-CRC to explore differences in factors associated with overall survival across different groups.

### Patients with or without mortality in different groups

Table 2 presents the differences in variables between patients EO-CRC who died during follow-up and those who survived. Patients who with mortality outcome had significantly more instances of a family history of colorectal cancer, low hemoglobin (HGB), higher carcinoembryonic antigen (CEA), CA19-9 (elevated cases), combined polyp, infiltrative type, a maximum cancer diameter of ≥5 cm, non-adenocarcinoma histology, distant metastasis, nodal metastasis, and higher TNM stage. On the other hand, the patients who survived after follow-up had significantly higher rates of being overweight, PMS2, and MSH6 expression.

**Table 2:** Patient”s clinical characteristics and results between patients with or without mortality in the aged <50 years group

Parameters	Survival (*n* = 169)	Dead (*n* = 78)	*P*
Sex			0.695
Male	93 (55.03%)	45 (57.69%)	
Female	76 (44.97%)	33 (42.31%)	
Age, year	43 (35.50, 47)	43.50 (36.75, 46)	0.788
Hypertension	22 (13.02%)	9 (11.54%)	0.744
Diabetes	15 (8.88%)	3 (3.85%)	0.157
Hyperlipidemia	6 (3.55%)	5 (6.41%)	0.311
Family history of colorectal cancer	8 (4.73%)	22 (28.21%)	<0.001
BMI, kg/m2	22.74 (20.31, 25.11)	22.27 (20.82, 24.17)	0.397
BMI group			0.055
Normal	85 (50.30%)	47 (60.26%)	
Underweight	16 (9.47%)	10 (12.82%)	
Overweight	51 (30.18%)	20 (25.64%)	
Obesity	17 (10.06%)	1 (1.28%)	
Overweight	68 (40.24%)	21 (26.92%)	0.043
HGB, g/L	126 (106.50, 140.50)	118 (92.50, 133)	0.037
Low HGB	54 (31.95%)	37 (47.44%)	0.019
CEA	2.31 (1.25, 5.29)	4.09 (1.62, 14.39)	0.003
CEA (+1 & log10 transformed)	0.52 (0.35, 0.80)	0.71 (0.42, 1.19)	0.003
Elevated CEA	52 (30.77%)	36 (46.15%)	0.019
CA19-9	8.54 (3.88, 18.59)	10.44 (4.41, 51.02)	0.072
CA19-9 (+1 & log10 transformed)	0.98 (0.69, 1.29)	1.06 (0.73, 1.71)	0.072
Elevated CA19-9	20 (11.83%)	21 (26.92%)	0.003
CA72-4	1.67 (0.98, 4.21)	2.17 (1.17, 4.67)	0.231
CA72-4 (+1 & log10 transformed)	0.43 (0.30, 0.72)	0.50 (0.34, 0.75)	0.231
Elevated CA72-4	31 (18.34%)	13 (16.67%)	0.749
Fecal occult blood	133 (78.70%)	58 (74.36%)	0.449
Combined polyp	75 (44.38%)	52 (66.67%)	0.001
Lesion			0.876
Rectum	14 (8.28%)	5 (6.41%)	
Left half	108 (63.91%)	51 (65.38%)	
Right half	47 (27.81%)	22 (28.21%)	
Pathological type			0.003
Ulcerative	54 (31.95%)	24 (30.77%)	
Polypoid	99 (58.58%)	34 (43.59%)	
Infiltrative	16 (9.47%)	20 (25.64%)	
Maximum diameter of cancer			<0.001
<5 cm	127 (75.15%)	37 (47.44%)	
≥5 cm	42 (24.85%)	41 (52.56%)	
Adenocarcinoma/high-grade epithelial neoplasia			<0.001
Yes	154 (91.12%)	58 (74.36%)	
No	15 (8.88%)	20 (25.64%)	
Differentiation			0.428
High	9 (5.33%)	2 (2.56%)	
Medium	122 (72.19%)	54 (69.23%)	
Low	38 (22.49%)	22 (28.21%)	
Distant metastasis	12 (7.10%)	30 (38.46%)	<0.001
Nodal metastasis	52 (30.77%)	45 (57.69%)	<0.001
TNM stage			<0.001
0–I–II	112 (66.27%)	22 (28.21%)	
III–IV	57 (33.73%)	56 (71.79%)	
Blood vessel			0.613
Negative	134 (79.29%)	64 (82.05%)	
Positive	35 (20.71%)	14 (17.95%)	
Lymphatic vessel			0.386
Negative	138 (81.66%)	60 (76.92%)	
Positive	31 (18.34%)	18 (23.08%)	
Nervous system			0.619
Negative	118 (69.82%)	52 (66.67%)	
Positive	51 (30.18%)	26 (33.33%)	
Her-2	92 (54.44%)	52 (66.67%)	0.070
MLH1	152 (89.94%)	67 (85.90%)	0.351
PMS2	152 (89.94%)	55 (70.51%)	<0.001
MSH2	157 (92.90%)	71 (91.03%)	0.607
MSH6	156 (92.31%)	60 (76.92%)	<0.001
BRAF V600E	18 (10.65%)	8 (10.26%)	0.925
PD-L1			0.821
≤10	108 (63.91%)	51 (65.38%)	
>10	61 (36.09%)	27 (34.62%)	
Ki-67			0.162
<80%	92 (54.44%)	35 (44.87%)	
≥80%	77 (45.56%)	43 (55.13%)	

Table 3 presents the results for LO-CRC. Compared to the survivors, the patients who died during follow-up had significantly older age, higher levels of CEA and CA19-9, more elevated cases of CA72-4, lesions on the left half, combined polyps, ulcerative type, longer maximum diameter of the cancer, non-adenocarcinoma histology, more distant metastases, higher TNM stage, positive rates of vascular and lymphatic invasion, more cases of BRAF V600E mutation, PD-L1 expression >10, and Ki-67 ≥80%. Conversely, those who died during follow-up also had lower BMI, fewer overweight cases, lower levels of HGB, and fewer cases with MLH1 and PMS2 expression.

**Table 3:** Patient’s clinical characteristics and results between patients with or without mortality in the aged ≥50 years group.

Parameters	Survival (*n* = 668)	Dead (*n* = 233)	*P*
Sex			0.779
Male	403 (60.33%)	143 (61.37%)	
Female	265 (39.67%)	90 (38.63%)	
Age, year	63 (57, 69)	68 (58, 77)	<0.001
Hypertension	189 (28.29%)	65 (27.90%)	0.908
Diabetes	96 (14.37%)	36 (15.45%)	0.688
Hyperlipidemia	23 (3.44%)	9 (3.86%)	0.766
Family history of colorectal cancer	34 (5.09%)	7 (3.00%)	0.188
BMI, kg/m2	23.00 (20.90, 24.95)	21.88 (19.89, 24.74)	<0.001
BMI group			<0.001
Normal	375 (56.14%)	127 (54.51%)	
Underweight	44 (6.59%)	35 (15.02%)	
Overweight	198 (29.64%)	56 (24.03%)	
Obesity	51 (7.63%)	15 (6.44%)	
Overweight	249 (37.28%)	71 (30.47%)	0.062
HGB, g/L	123 (106, 135)	111 (90, 125)	<0.001
Low HGB	228 (34.13%)	143 (61.37%)	<0.001
CEA	3.19 (1.78, 8.09)	4.19 (2.12, 12.04)	0.016
CEA (+1 & log10 transformed)	0.62 (0.44, 0.96)	0.72 (0.49, 1.12)	0.016
Elevated CEA	249 (37.28%)	100 (42.92%)	0.128
CA19-9	8.16 (4.10, 18.36)	14.85 (4.64, 43.40)	<0.001
CA19-9 (+1 & log10 transformed)	0.96 (0.71, 1.29)	1.20 (0.75, 1.65)	<0.001
Elevated CA19-9	74 (11.08%)	70 (30.04%)	<0.001
CA72-4	1.87 (1.01, 4.29)	2.35 (1.14, 5.05)	0.068
CA72-4 (+1 & log10 transformed)	0.46 (0.30, 0.72)	0.53 (0.33, 0.78)	0.068
Elevated CA72-4	90 (13.47%)	44 (18.88%)	0.046
Fecal occult blood	558 (83.53%)	196 (84.12%)	0.835
Combined polyp	266 (39.82%)	129 (55.36%)	<0.001
Lesion			<0.001
Rectum	77 (11.53%)	29 (12.45%)	
Left half	432 (64.67%)	115 (49.36%)	
Right half	159 (23.80%)	89 (38.20%)	
Pathological type			<0.001
Ulcerative	249 (37.28%)	114 (48.93%)	
Polypoid	396 (59.28%)	102 (43.78%)	
Infiltrative	23 (3.44%)	17 (7.30%)	
Maximum diameter of cancer			<0.001
<5 cm	431 (64.52%)	116 (49.79%)	
≥5 cm	237 (35.48%)	117 (50.21%)	
Adenocarcinoma/high-grade epithelial neoplasia			<0.001
Yes	587 (87.87%)	184 (78.97%)	
No	81 (12.13%)	49 (21.03%)	
Differentiation			0.763
High	24 (3.59%)	7 (3.00%)	
Medium	552 (82.63%)	190 (81.55%)	
Low	92 (13.77%)	36 (15.45%)	
Distant metastasis	94 (14.07%)	58 (24.89%)	<0.001
Nodal metastasis	226 (33.83%)	91 (39.06%)	0.151
TNM stage			0.003
0–I–II	349 (52.25%)	95 (40.77%)	
III–IV	319 (47.75%)	138 (59.23%)	
Blood vessel			<0.001
Negative	562 (84.13%)	148 (63.52%)	
Positive	106 (15.87%)	85 (36.48%)	
Lymphatic vessel			0.001
Negative	570 (85.33%)	177 (75.97%)	
Positive	98 (14.67%)	56 (24.03%)	
Nervous system			0.103
Negative	416 (62.28%)	131 (56.22%)	
Positive	252 (37.72%)	102 (43.78%)	
Her-2	403 (60.33%)	145 (62.23%)	0.608
MLH1	654 (97.90%)	205 (87.98%)	<0.001
PMS2	648 (97.01%)	210 (90.13%)	<0.001
MSH2	650 (97.31%)	226 (97.00%)	0.804
MSH6	656 (98.20%)	224 (96.14%)	0.072
BRAF V600E	49 (7.34%)	69 (29.61%)	<0.001
PD-L1			<0.001
≤10	506 (75.75%)	146 (62.66%)	
>10	162 (24.25%)	87 (37.34%)	
Ki-67			<0.001
<80%	393 (58.83%)	83 (35.62%)	
≥80%	275 (41.17%)	150 (64.38%)	

### LASSO results

**Figure 2:**
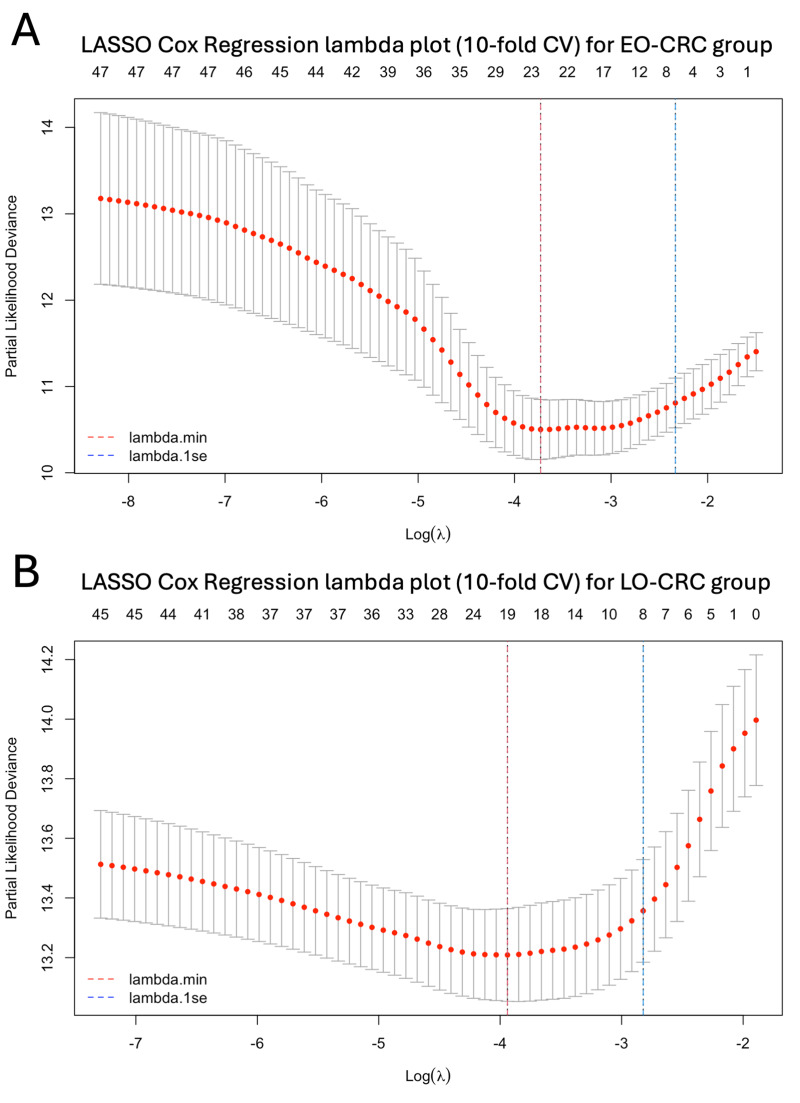
LASSO Cox regression lambda plot for the two age groups (<50 years and ≥50 years). For EO-CRC group, the log(lambda.min) is −3.73 and the log(lambda.1se) is −2.41; for LO-CRC group, the log(lambda.min) is −3.94 and the log(lambda.1se) is −2.26.

**Figure 3:**
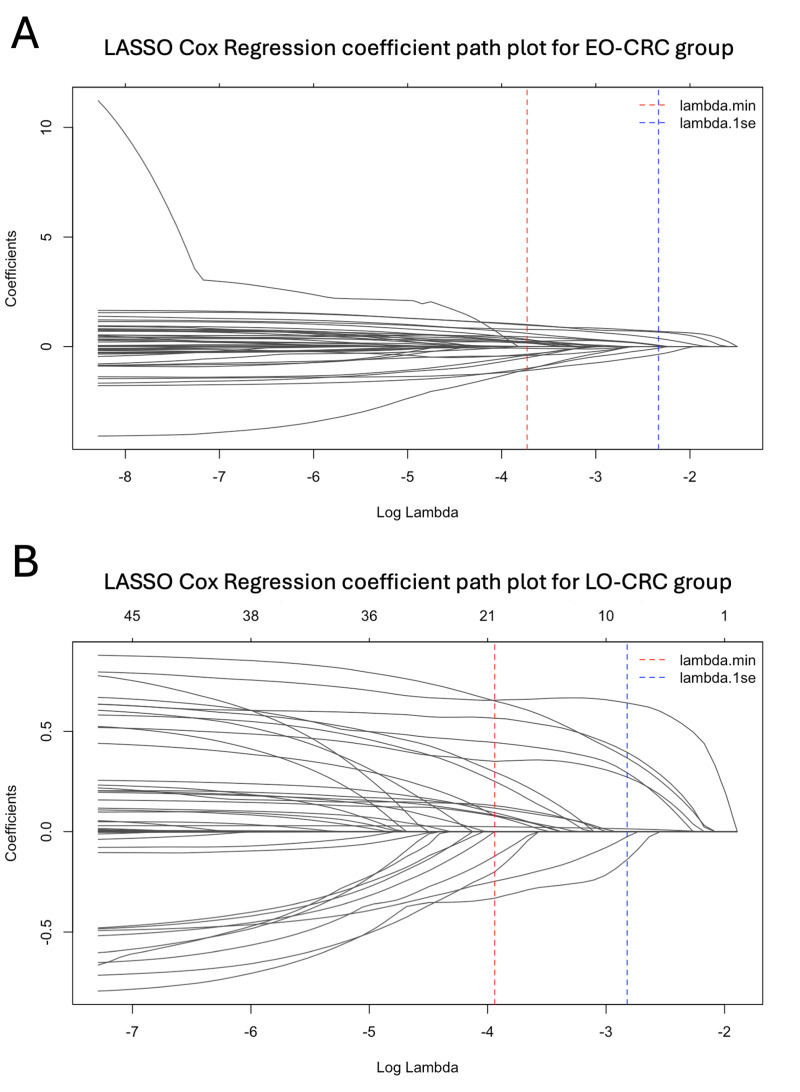
LASSO Cox regression coefficient path plot for the two age groups (<50 years and ≥ 50 years). The vertical blue line segment at log(lambda1se) intersects the coefficient lines, indicating that the corresponding independent variables have non-zero LASSO coefficients, which means that these variables have a significant association with the Cox outcome.

Figure 2 presents the lambda results of the LASSO analysis for all variables across two groups, For EO-CRC group, the log(lambda.min) is −3.73 and the log(lambda.1se) is −2.41; for LO-CRC group, the log(lambda.min) is −3.94 and the log(lambda.1se) is −2.26. The 1 S.E. criterion was used. Figure 3 illustrates the coefficient convergence process for each variable. The vertical line segment at log(lambda.1se) intersects the coefficient lines, indicating that the corresponding independent variables have non-zero LASSO coefficients, which means that these variables have a significant association with the Cox outcome. Finally, Table 4 displays the variables with non-zero coefficients, identified as being associated with OS, in both groups.

**Table 4:** LASSO results for each age group, the variables with non-zero coefficients related to OS.

	EO-CRC			LO-CRC	
No	Parameters	Absolute value of the LASSO coefficient		Parameters	Absolute value of the LASSO coefficient
1	Distant metastasis	0.6959		BRAF V600E	0.6418
2	Family history of colorectal cancer	0.6582		Elevated CA19-9	0.3956
3	TNM stage	0.3688		Ki-67	0.3676
4	PMS2	−0.3591		Low HGB	0.2937
5	MSH6	−0.0780		Blood vessel (positive)	0.2723
6	Maximum diameter of cancer	0.0582		MLH1	−0.1389
7	Combined polyp	0.0496		Pathological type	−0.0251
8	Ki-67	0.0313		Age	0.0140

**Notes.**

Sort by the absolute value of the coefficients from largest to smallest.

In EO-CRC, the eight variables significantly associated with overall survival (OS) are ranked in descending order according to their LASSO coefficients as follows: distant metastasis, family history of colorectal cancer, TNM stage, PMS2, MSH6, maximum diameter of cancer, combined polyp, and Ki-67. In LO-CRC, the variables significantly associated with overall survival (OS), in order, are BRAF V600E, elevated CA19-9, Ki-67, low hemoglobin (HGB), blood vessel invasion (positive), MLH1, pathological type, and age.

Among the two groups, only Ki-67 is the intersecting variable. Preliminarily, it appears that the key risk factors vary greatly across different groups.

### The multivariate model in predicting OS

The variables selected by LASSO were further incorporated into multivariate Cox proportional hazards regression models, and the results are presented in Table 5. All variables remained significant. The magnitude of the hazard ratio (HR) can be interpreted to explain the risk or protective nature of the variables.

**Table 5:** The multivariate Cox proportional hazards regression models in predicting patient’s OS.

Parameters	Adjusted HR (95% CI)	*P*
**Model: Age group <50 years (EO-CRC)**		
Distant metastasis	2.52 (1.38 to 4.61)	0.003
Family history of colorectal cancer	2.17 (1.20 to 3.93)	0.010
TNM stage		
0–I–II	1	–
III–IV	2.86 (1.55 to 5.29)	<0.001
PMS2	0.35 (0.19 to 0.63)	<0.001
MSH6	0.55 (0.31 to 0.95)	0.033
Maximum diameter of cancer		
<5 cm	1	–
≥5 cm	1.96 (1.22 to 3.15)	0.005
Combined polyp	1.82 (1.12 to 2.93)	0.015
Ki-67		
<80%	1	–
≥80%	2.09 (1.27 to 3.43)	0.004
**Model: Age group ≥50 years (LO-CRC)**		
BRAF V600E	2.03 (1.45 to 2.84)	<0.001
Elevated CA19-9	1.90 (1.39 to 2.59)	<0.001
Ki-67		
<80%	1	–
≥80%	2.09 (1.58 to 2.77)	<0.001
Low HGB	1.98 (1.51 to 2.59)	<0.001
Blood vessel (positive)	1.63 (1.20 to 2.20)	0.002
MLH1	0.59 (0.39 to 0.90)	0.015
Pathological type		0.022
Ulcerative	1	–
Polypoid	0.68 (0.52 to 0.89)	0.006
Infiltrative	0.81 (0.48 to 1.39)	0.452
Age, year	1.03 (1.01 to 1.04)	<0.001

In EO-CRC, distant metastasis, family history, higher TNM stage, longer maximum diameter of cancer, combined polyp, and a Ki-67 ≥80% are all associated with an increased risk of long-term mortality. Meanwhile, PMS2 and MSH6 are protective factors.

In LO-CRC, BRAF V600E mutation, elevated CA19-9, KI-67 ≥80%, low hemoglobin (HGB), positive result of blood vessel invasion, and older age are all risk factors for long-term mortality. Meanwhile, MLH1 and polypoid type (compared to ulcerative type) are protective factors.

**Figure 4:**
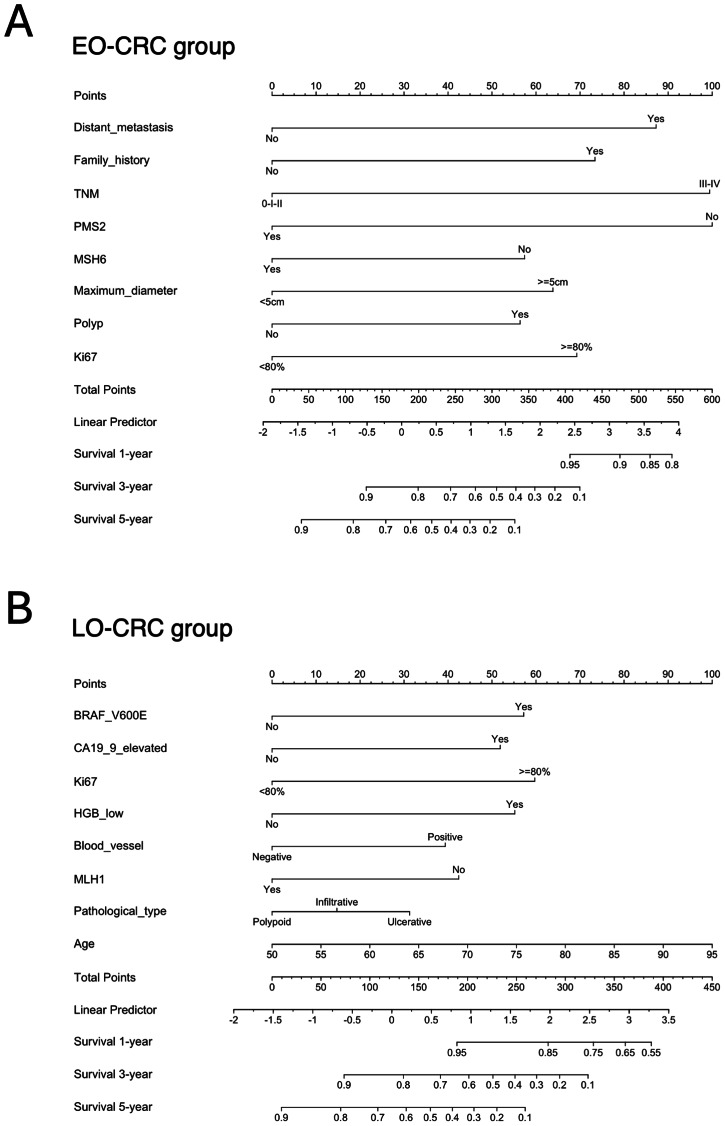
Nomogram derived from the multivariate Cox regression models for the two age groups (<50 years and ≥ 50 years).

Figure 4 are the nomograms of these two corresponding multivariate models. The nomogram provides an intuitive tool for individualized risk prediction. For a given patient, each predictor corresponds to a specific point value on the scale. The total points are calculated by summing the points for all predictors, which can then be mapped to the predicted probability of survival at specific time points.

### The validation of predictive models

The C-index (S.E.) for the multivariate Cox model for EO-CRC groupand LO-CRC group are 0.829 (0.023) and 0.751 (0.018), respectively, both demonstrating good predictive power.

**Figure 5:**
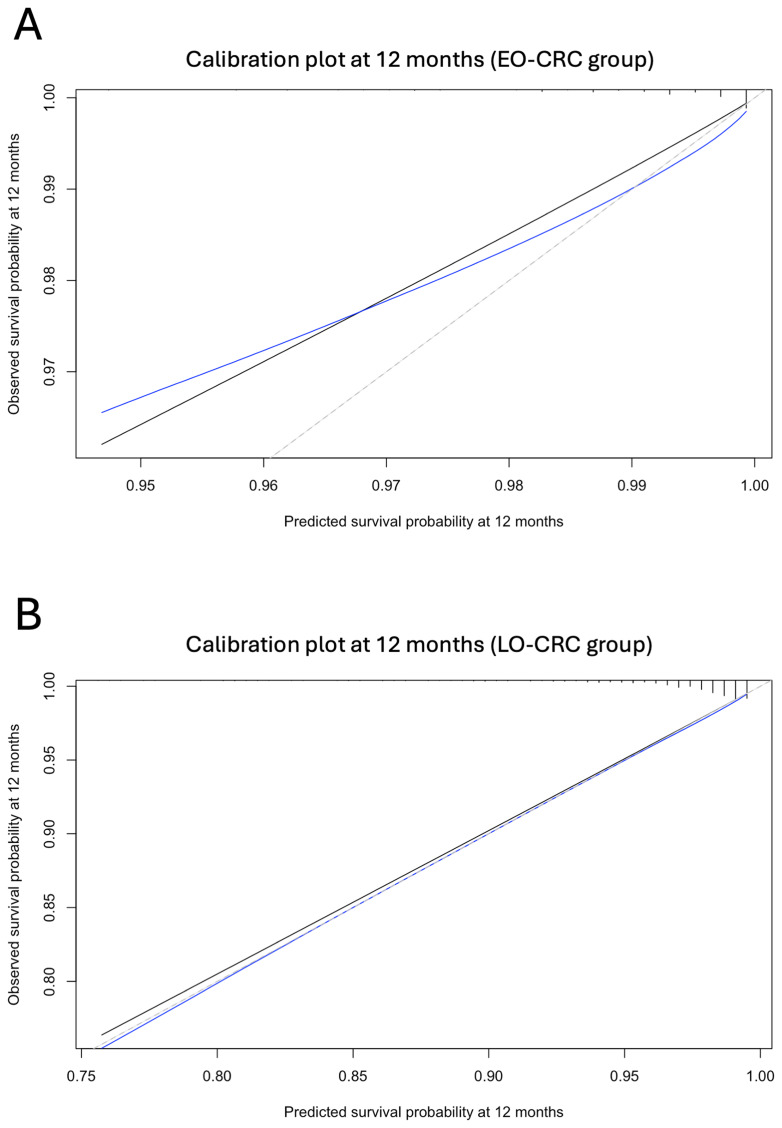
One-year calibration plots of the prediction models (nomograms) for the two age groups (<50 years and ≥ 50 years).

As shown in Fig. 5, taking the 1-year predictive power as an example, the calibration plots for both groups are very close to the 45-degree diagonal line, except that the predicted values for the EO-CRC groupare slightly higher than the actual risk.

**Figure 6:**
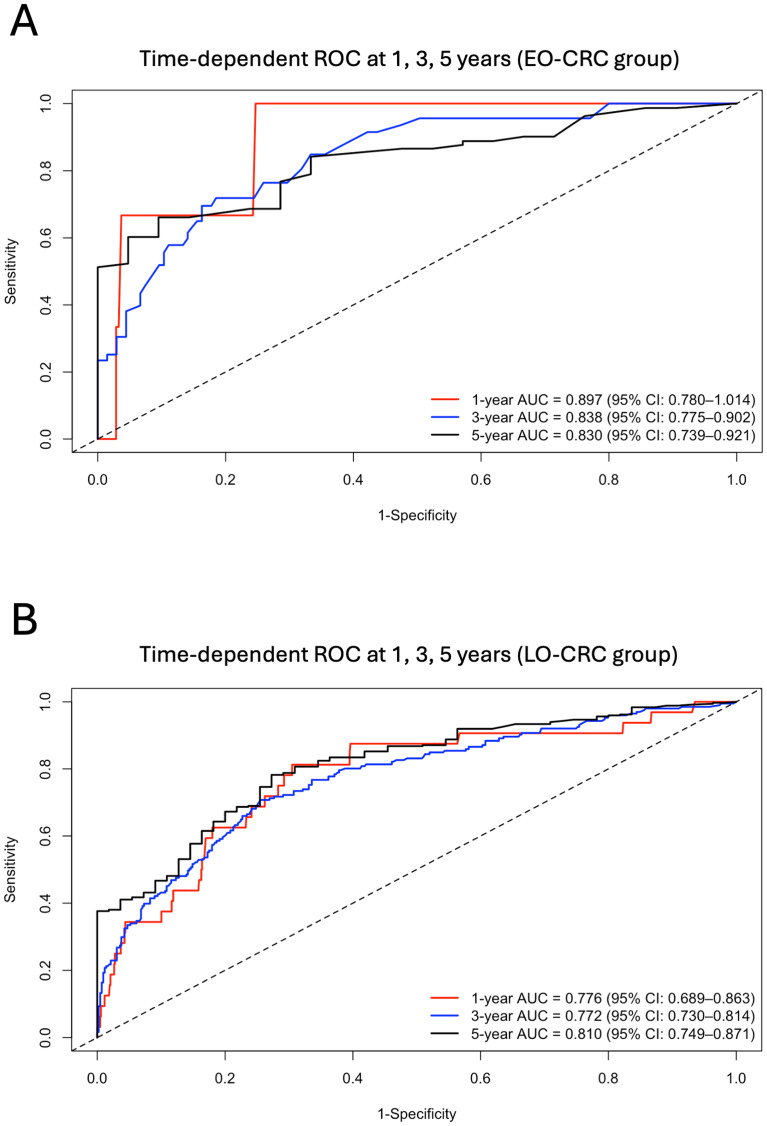
Time-dependent ROC curves and AUC results for the two age groups (<50 years and ≥ 50 years).

Figure 6 presents the time-dependent ROC and AUC results for two groups, with outcomes that are very favorable. In the EO-CRC group, the AUCs for 1, 3, and 5 years all exceed 0.80, with the first year even approaching 0.90; in the LO-CRC group, the AUCs for 1 and 3 years are slightly below 0.80 (0.78 and 0.77), while the predictive power for the fifth year is an AUC of 0.81. All this evidence supports the model’s good predictive capability. We performed statistical comparisons between time points. The results showed that the confidence intervals of the AUCs overlapped, indicating no statistically significant differences among the 1-, 3-, and 5-year AUCs.

For prognostic modeling with binary outcome, the adequacy of sample size is commonly evaluated based on the number of outcome events relative to the number of predictors included in the model (events per variable, EPV). In the EO-CRC group, there were 78 events with eight variables included in the final multivariable Cox model (EPV ≈ 9.75), which is close to the commonly recommended threshold of 10 events per variable. In the LO-CRC group, the number of events was substantially larger (233 events for eight variables), providing more stable estimates.

### Variable importance by random survival forest analysis

Compared to LASSO analysis, which assumes a default linear relationship, random forest takes into account the nonlinear relationships between independent variables and outcomes. By using this second machine learning method for ranking, we can validate and observe the importance of variables related to patient’s OS.

**Figure 7:**
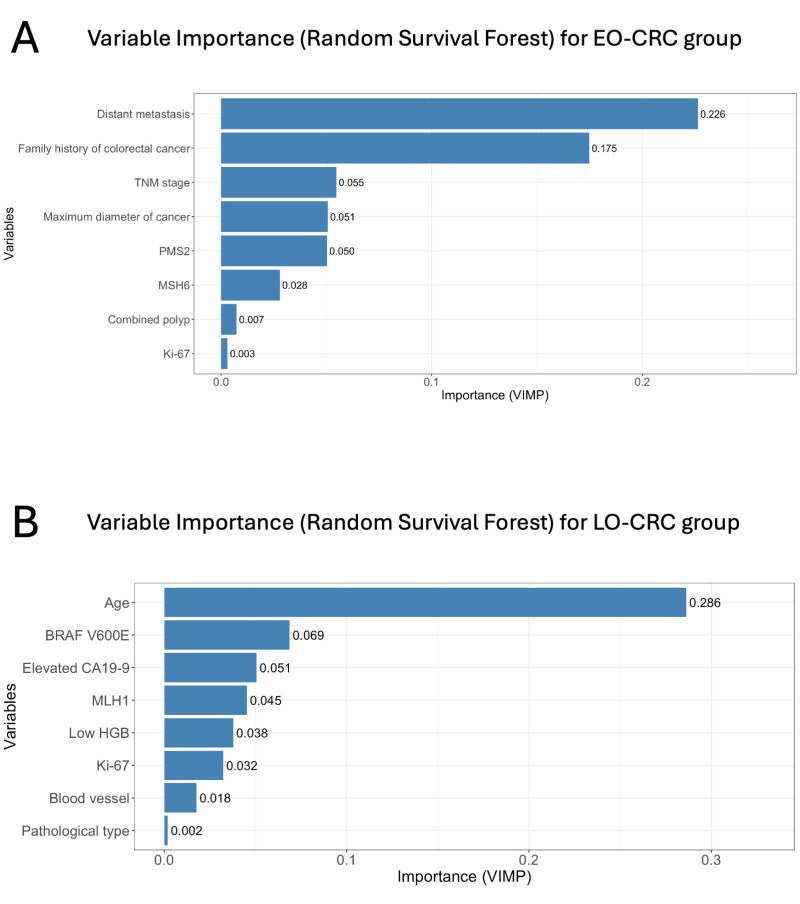
Variable importance rankings based on the random survival forest models for the two age groups (<50 years and ≥ 50 years).

When comparing the results of Fig. 7 with those of the LASSO, in the EO-CRC group, the variable importance remains largely unchanged except for a slight increase in the maximum diameter of cancer. The two most important variables are distant metastasis and family history of colorectal cancer. In the LO-CRC group, the importance of age dramatically jumps from the last to the first position, while the order of the other variables remains similar. According to the results of the random forest, the two most critical variables are age and BRAF V600E.

## Discussion

This study integrated traditional statistical methods with machine learning algorithms to construct age-specific prognostic models with excellent predictive performance (the C-indices of the EO-CRC and LO-CRC models were 0.829 and 0.751, respectively). The analysis revealed fundamental differences in the prognostic determinants between the two patient groups, profoundly reflecting the etiological and pathogenic mechanisms underlying early-onset and late-onset colorectal cancer. Overall, the risk characteristics of EO-CRC are more indicative of genetic susceptibility and intrinsic aggressive biological behavior, whereas LO-CRC is more closely associated with age-related acquired molecular alterations, chronic metabolic diseases, and long-term environmental exposures. Our findings extend prior research by integrating traditional statistics with machine learning to construct age-specific prognostic models in a Chinese cohort—an approach rarely applied in this context. The dual-analytic strategy confirms key predictors while capturing non-linear relationships.

This study found that the elevation of CEA in LO-CRC was significantly higher than that in EO-CRC, which may be primarily related to increased tumor burden and later disease stage. Elevated CEA levels in advanced colorectal cancer are associated with poorer survival prognosis (Aggarwal et al., 2013). In this study, the proportion of late-stage TNM staging in LO-CRC was higher than that in EO-CRC, although there was no statistical difference.

At the molecular level, this difference primarily stems from distinct carcinogenic pathways. This study indicates that the proportion of individuals with a family history of colorectal cancer in EO-CRC is significantly higher than that in LO-CRC (*p* < 0.001). Compared to the general population, individuals with a family history of colorectal cancer are advised to undergo colorectal cancer screening earlier (Boardman et al., 2020). Furthermore, EO-CRC exhibits a higher proportion of DNA Mismatch Repair Deficiency (dMMR) (particularly deletions of PMS2 and MSH6) and PD-L1 expression, which is closely associated with genetic syndromes, particularly Lynch syndrome. The typical mechanism of Lynch syndrome involves germline mutations in MMR genes (such as MLH1, MSH2, MSH6, PMS2, or EPCAM) leading to defective dMMR and high-frequency microsatellite instability (MSI-H) (Pathak et al., 2018). The above-mentioned gene mutations result in the inability of cells to recognize and repair spontaneous mutations (dMMR), leading to a very high burden of tumor mutations and alterations in microsatellite sequences, making these tumors highly microsatellite unstable (MSI-H) (André et al., 2020). Therefore, in this study, PMS2 and MSH6 positivity (no mutation) were protective factors for EO-CRC. Some studies suggest that hereditary colorectal cancer accounts for approximately 5% to 10% of the overall cases (Eng et al., 2022; Salvatore et al., 2020), with approximately 2–4% of patients harboring Lynch syndrome (Yu, Ding & Jiang, 2023). Patients with Lynch syndrome have a 2- to 3-fold increased likelihood of developing early-onset colorectal cancer, and the younger the onset age, the greater the likelihood of hereditary colorectal cancer (Eng et al., 2022). This results in a highly immunogenic tumor microenvironment, aligning closely with previous research indicating that patients with dMMR and MSI-H are suitable candidates for first-line treatment with immune checkpoint inhibitors (Sinicrope, 2022). This explains why such tumors are sensitive to immunotherapy, and family history emerges as one of the strongest prognostic predictors. Since most patients with Lynch syndrome remain undiagnosed, they are unaware of their high cancer risk. Current guidelines recommend MMR and MSI testing for all newly diagnosed colorectal cancers to detect Lynch syndrome (Sinicrope, 2022). The average age of colorectal cancer caused by Lynch syndrome is 45 years old, so some studies suggest that patients with Lynch syndrome should undergo colonoscopy screening at the age of 25 and repeat it every 1–2 years (Pathak et al., 2018).

Conversely, the risk factors in LO-CRC are BRAF V600E mutations and MLH1 deletions. LO-CRC patients include a higher proportion of sporadic MSI-H tumors caused by MLH1 hypermethylation and frequent BRAF V600E mutations, representing a sporadic carcinogenic pathway (Sinicrope, 2022), previous studies have found that colorectal cancer patients carrying BRAF V600E mutations are typically older and more likely to be female (Corcoran et al., 2018), often accompanied by lymph node and peritoneal metastasis (Sinicrope et al., 2016; Adackapara et al., 2013), the median overall survival for patients with advanced-stage BRAF V600E mutations is approximately 9 months (Ruiz-Saenz et al., 2023), whereas wild-type patients can survive for over 20 months. The National Comprehensive Cancer Network (NCCN) guidelines for 2025 state that BRAF mutations are a strong prognostic marker (Gradishar et al., 2025).

In terms of clinical pathological characteristics, the differences in the aforementioned molecular mechanisms further manifest as distinct disease phenotypes. EO-CRC exhibits more concurrent polyps, a higher prevalence of invasive lesions, and more common low differentiation, among other aggressive histological features. Its distant metastasis and TNM staging are key prognostic factors, which may be attributed to the rapid progression caused by driver gene mutations. The higher incidence of aggressive histological features in EO-CRC is consistent with previous research findings (Moon et al., 2023). Simultaneously, the significant familial aggregation suggests a potential genetic background. The higher incidence of hypertension, diabetes, ulcerative lesions, and positive fecal occult blood in LO-CRC patients may reflect the process of chronic, ulcerative lesions developing in the mucosa under the influence of long-term environmental factors (such as diet and metabolism). Ulcerative lesions are more prone to cause bleeding, and the subsequent low hemoglobin level also becomes an independent risk factor.

Several limitations should be acknowledged. First, this was a single-center retrospective study, which may introduce selection bias. Second, the proportion of EO-CRC patients was relatively low (247/1,148), which may reduce statistical power for detecting prognostic factors specific to this subgroup. Third, the model was not validated in an external cohort. Future studies should aim to validate our findings in multicenter cohorts and through prospective study designs. Additionally, the inclusion of comprehensive genetic ((such as APC, mutY DNA glycosylase (MUTYH), Bone morphogenetic protein receptor, type IA (BMPR1A), SMAD family member 4 (SMAD4), phosphatase and tensin homolog (PTEN), Serine-Threonine Kinase 11 (STK11), and other polyposis genes (Sinicrope, 2022)) and environmental exposure data will be essential to further refine risk stratification.

In summary, this study suggests that EO-CRC and LO-CRC have fundamentally different prognostic determinants. The EO-CRC model emphasizes genetic susceptibility and tumor invasiveness, indicating that this group of patients may benefit from early genetic counseling, MMR/MSI testing, and immune checkpoint inhibitor therapy. For example, individuals under the age of 50 with a family history of colorectal cancer or a history of polyps should undergo colonoscopy screening earlier. In contrast, the LO-CRC model highlights age, acquired molecular changes, and chronic systemic factors, supporting the inclusion of metabolic and geriatric assessments in routine tumor care. For example, those over 50 years old with elevated CA19-9 or decreased hemoglobin should strengthen colorectal cancer screening. These findings provide a foundation for age specific stratified management and age based personalized treatment strategies in clinical practice. Future research should validate the model in multi center cohorts, increase sample size, combine multi omics data with prospective designs, further refine the etiology and prognosis stratification of different age subgroups, and further combine detailed genetic susceptibility gene testing (such as adenomatous polyposis Escherichia coli (APC), MUTHH, *etc.*) and long-term environmental exposure data to more comprehensively elucidate the etiology network of these two types of cancer, providing a foundation for precise prevention and early intervention.

##  Supplemental Information

10.7717/peerj.21484/supp-1 Supplemental Information 1 Data

10.7717/peerj.21484/supp-2 Supplemental Information 2 Variable missing rate

10.7717/peerj.21484/supp-3 Supplemental Information 3 STROBE checklist

## Abbreviations

EO-CRC: Early-onset colorectal cancer
LO-CRC: Late-onset colorectal cancer
CRC: Colorectal cancer
JNCC: Journal of the National Cancer Center
PMS2: PMS1 homolog 2, mismatch repair system component
MLH1: MutL Homolog 1
MSH2: MutS Homolog 2
MSH6: MutS Homolog 6
EPCAM: Epithelial Cell Adhesion Molecule
BARF V600E: BRAF gene V600E mutation
Ki-67: Nuclear-associatedantigenki-67
PD-L1: Programmed Cell Death Ligand 1
TNM: Tumor Node Metastasis classification
CA: A Cancer Journal for Clinicians
BMI: Body Mass Index
CEA: Carcinoembryonic antigen
CA19-9: Carbohydrate Antigen 19-9
CA72-4: Carbohydrate Antigen 72-4
HGB: Hemoglobin
Her-2: Human Epidermal Growth Factor Receptor 2
OS: Overall survival
dMMR: DNA Mismatch Repair Deficiency
MSI-H: Microsatellite Instability-High
NCCN: The National Comprehensive Cancer Network
APC: Adenomatous Polyposis Coli
MUTYH: mutY DNA glycosylase
BMPR1A: Bone morphogenetic protein receptor, type IA
SMAD4: SMAD family member 4
PTEN: Phosphatase and tensin homolog
STK11: Serine-Threonine Kinase 11

## Additional Information and Declarations

### Data Availability

The following information was supplied regarding data availability: The raw data is available in the Supplemental File.
