# Supplementary figures and images for: A retrospective study of differential prognostic factors in early-onset versus late-onset colorectal cancer: a comprehensive clinical and machine learning analysis
Source: PeerJ. 2026 Jul 1;14:e21484. doi: 10.7717/peerj.21484 (PMC13332719; doi:10.7717/peerj.21484)

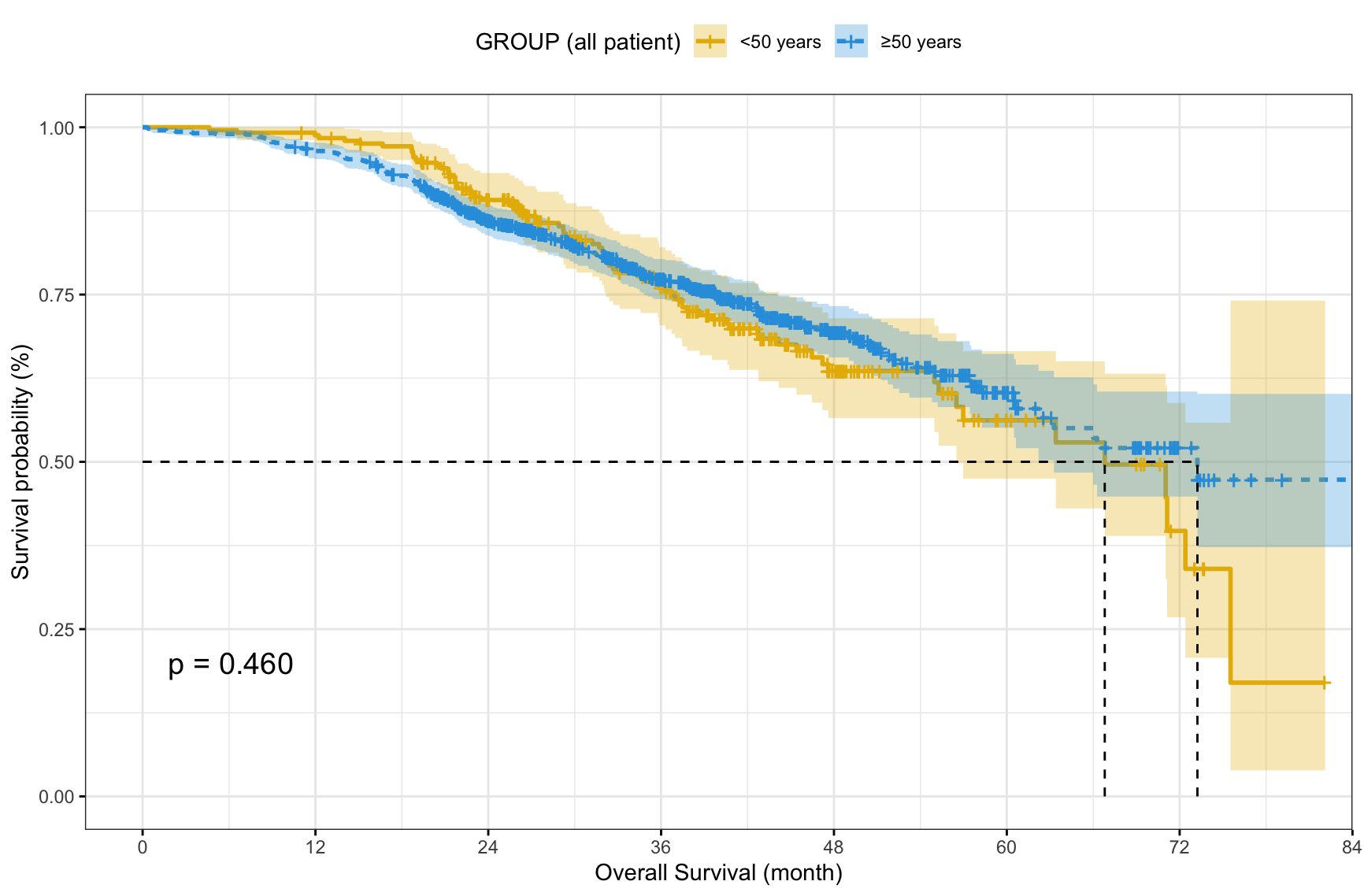

Supplement: Supplemental Information 1 [file peerj-14-21484-s001.zip › Figures/Figure 1/Figure 1.png]

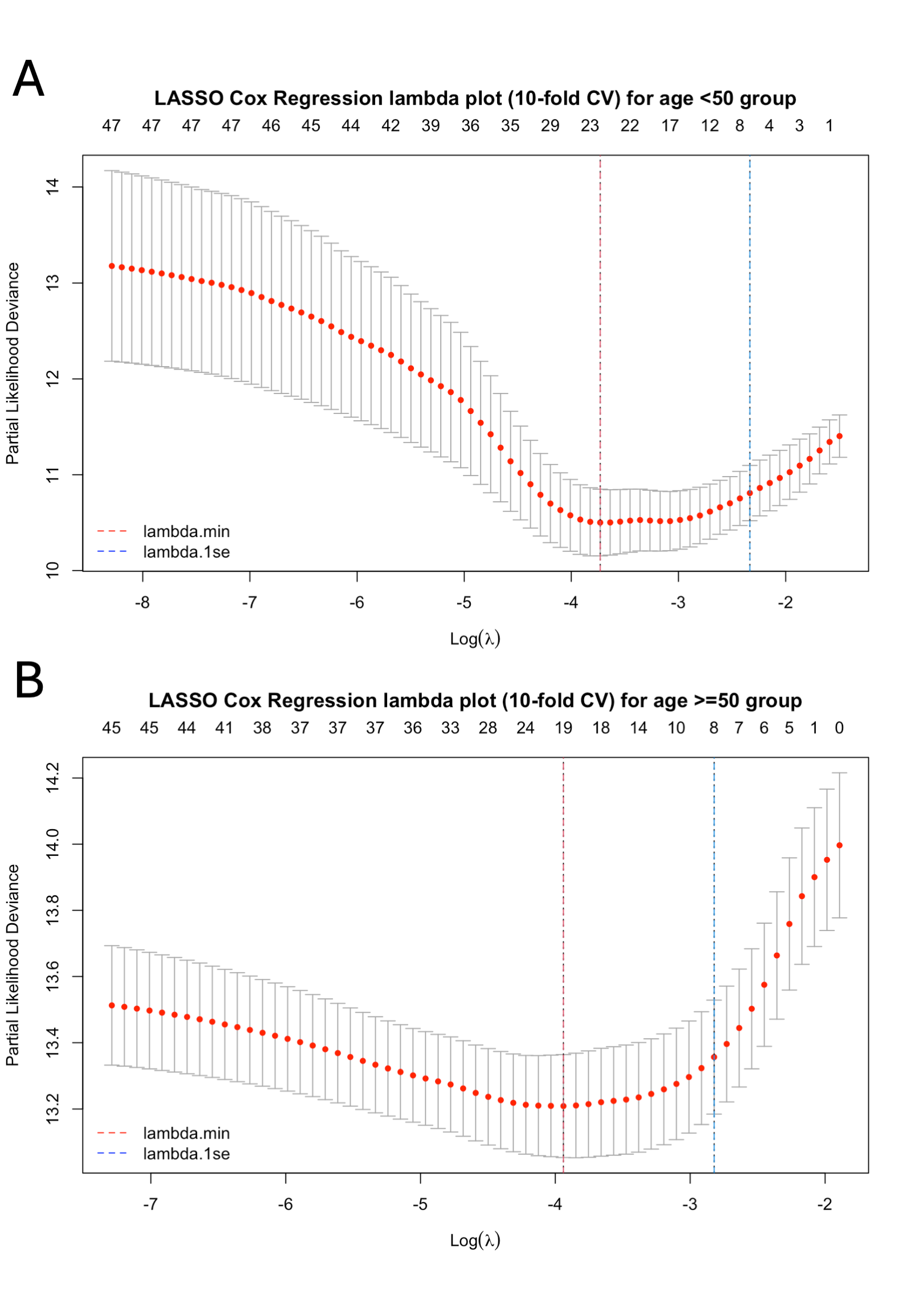

Supplement: Supplemental Information 1 [file peerj-14-21484-s001.zip › Figures/Figure 2/Figure 2.png]

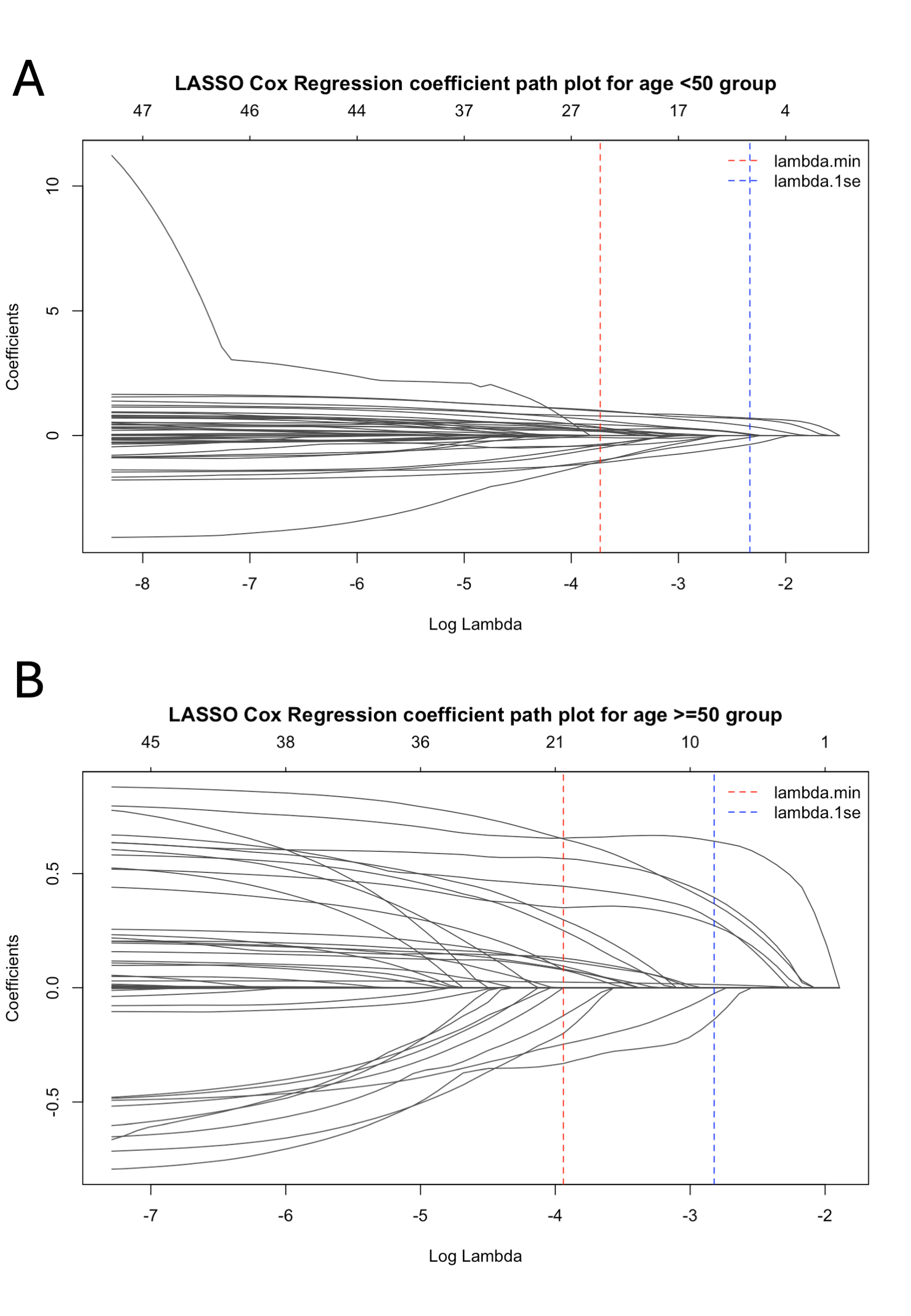

Supplement: Supplemental Information 1 [file peerj-14-21484-s001.zip › Figures/Figure 3/Figure 3.png]

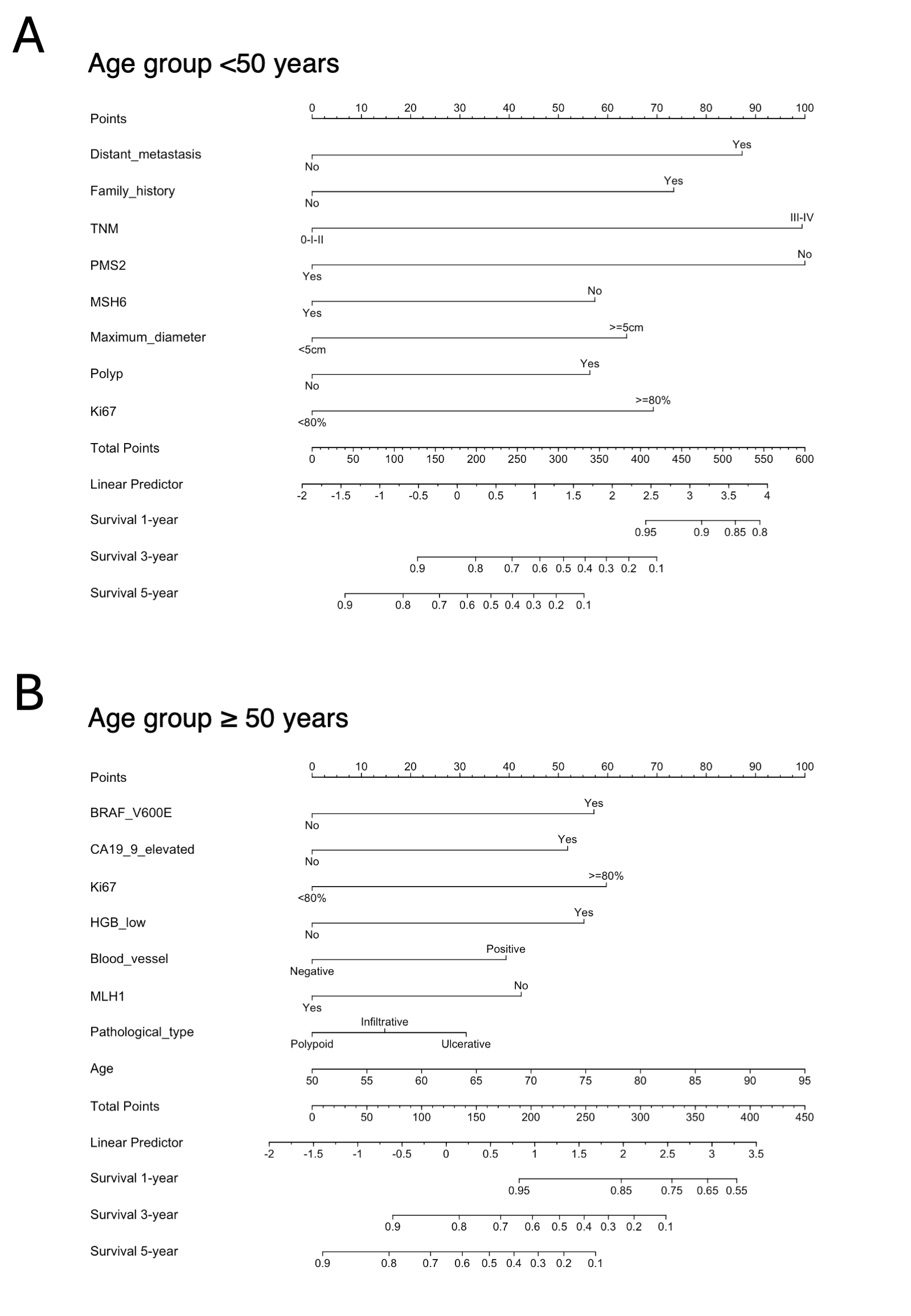

Supplement: Supplemental Information 1 [file peerj-14-21484-s001.zip › Figures/Figure 4/Figure 4.png]

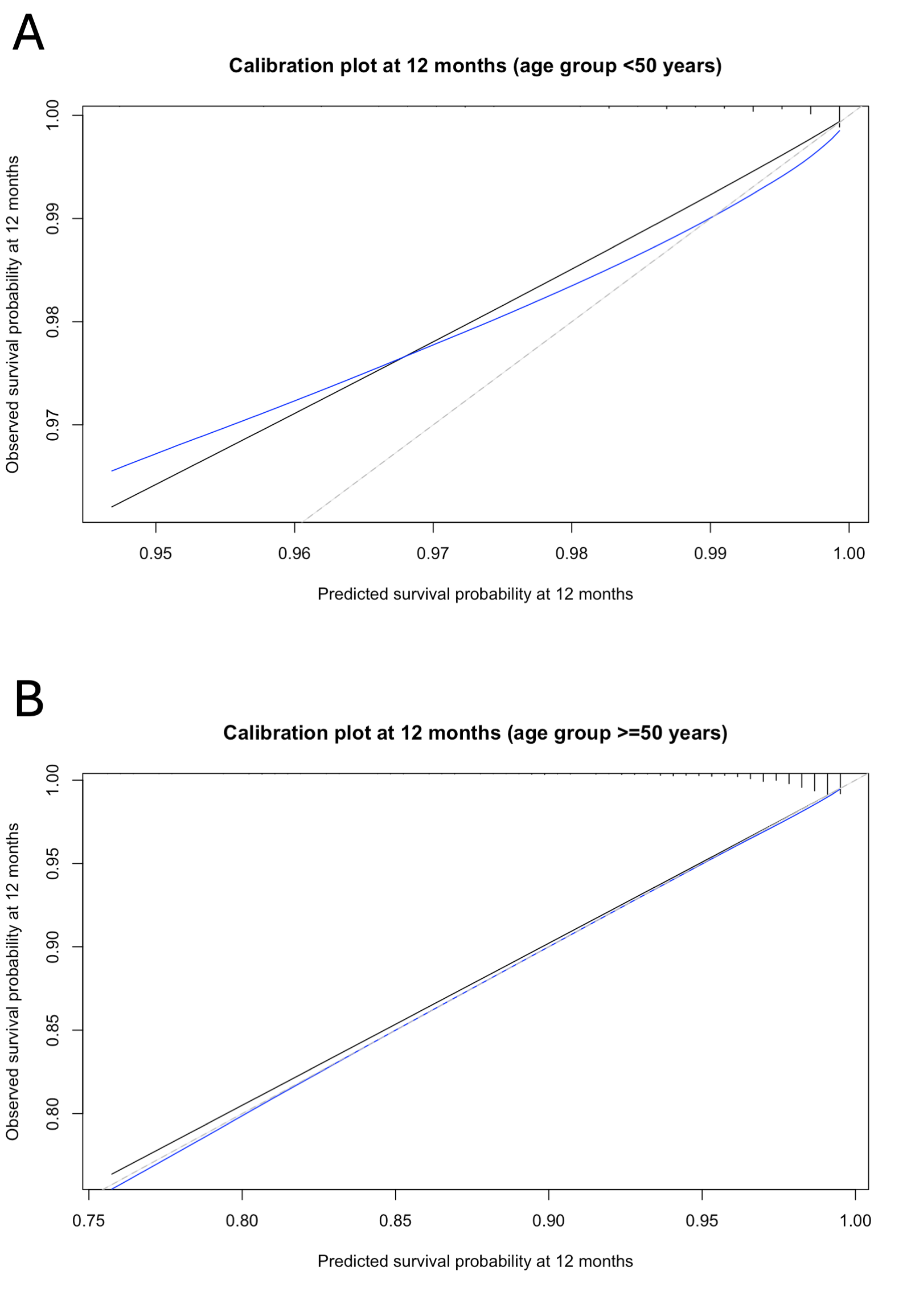

Supplement: Supplemental Information 1 [file peerj-14-21484-s001.zip › Figures/Figure 5/Figure 5.png]

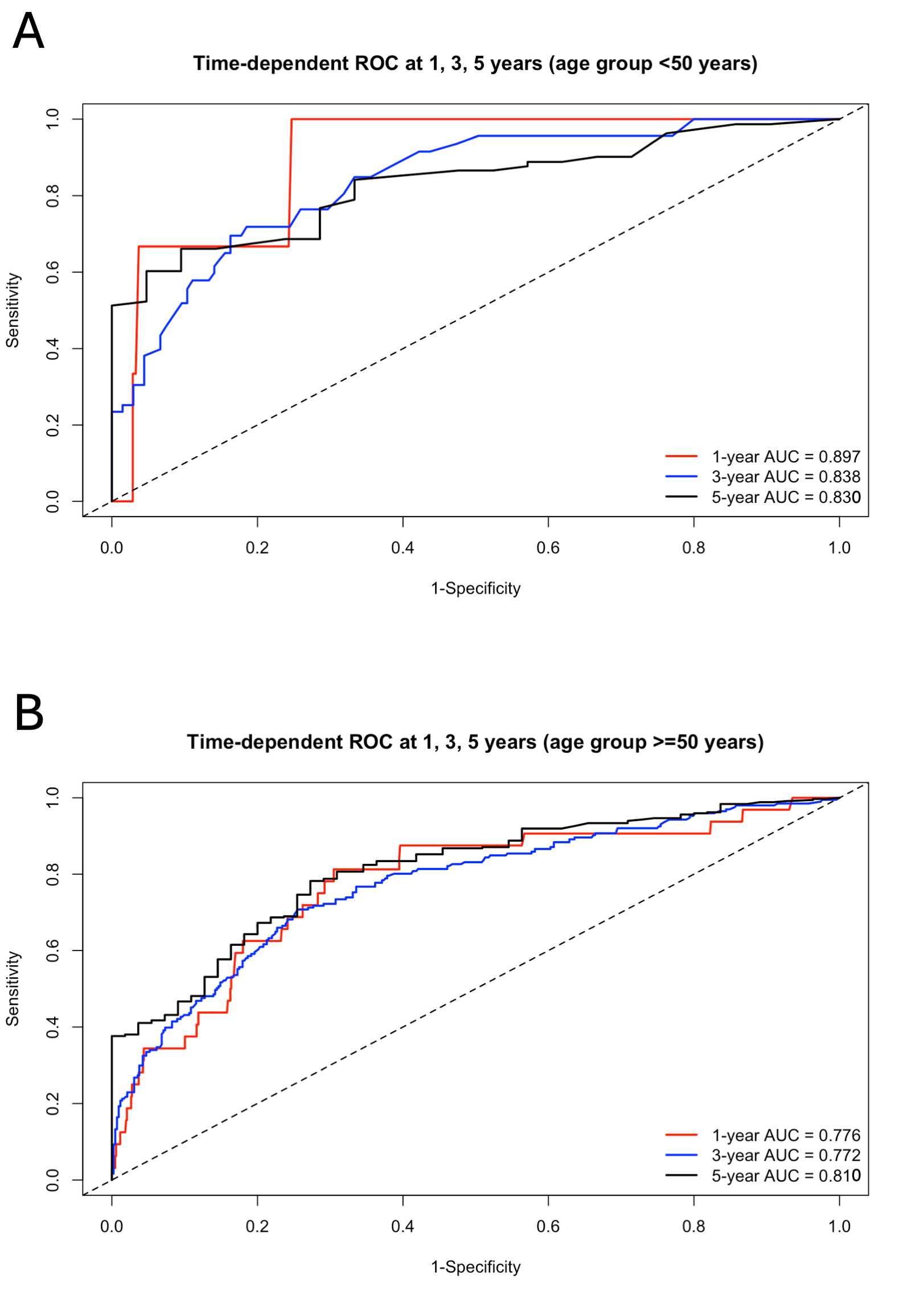

Supplement: Supplemental Information 1 [file peerj-14-21484-s001.zip › Figures/Figure 6/Figure 6.png]

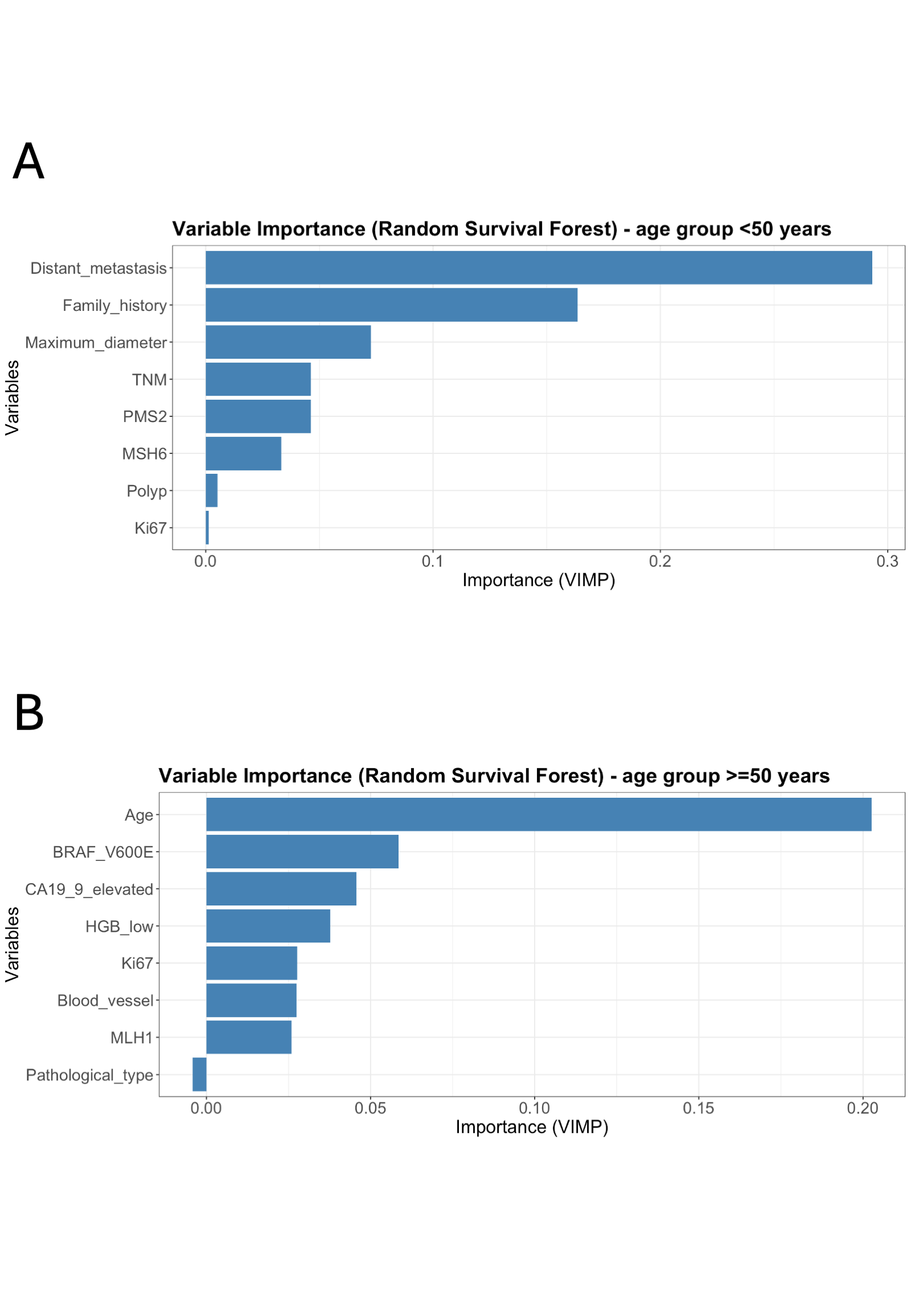

Supplement: Supplemental Information 1 [file peerj-14-21484-s001.zip › Figures/Figure 7/Figure 7.png]
