# Supplementary material for: A retrospective study of differential prognostic factors in early-onset versus late-onset colorectal cancer: a comprehensive clinical and machine learning analysis
Source: PeerJ. 2026 Jul 1;14:e21484. doi: 10.7717/peerj.21484 (PMC13332719; doi:10.7717/peerj.21484)
Supplement: Supplemental Information 2 [file peerj-14-21484-s002.docx]

**Supplementary Table S1. V ariable missing rate.**

| Variables | Missing rate |
| --- | --- |
| CEA | 10.66% |
| CA19-9 | 10.57% |
| CA72-4 | 10.74% |
| Elevated CEA | 10.74% |
| Elevated CA19-9 | 10.66% |
| Elevated CA72-4 | 10.83% |
| Fecal occult blood | 15.37% |
| Combined polyp | 12.84% |
| Lesion | 4.45% |
| Adenocarcinoma | 0.70% |
| Blood vessel | 2.62% |
| Lymphatic vessel | 2.71% |
| Nervous system | 2.71% |
| Her-2 | 9.34% |
| MLH1 | 9.17% |
| PMS2 | 9.17% |
| MSH2 | 9.17% |
| MSH6 | 9.08% |
| BRAF-V600E | 8.73% |
| PD-L1 | 16.77% |
| Ki-67 | 9.17% |
